# LinearCapR: linear-time computation of per-nucleotide structural-context probabilities of RNA without base-pair span limits

**DOI:** 10.1093/bioinformatics/btag295

**Published:** 2026-06-15

**Authors:** Takumi Otagaki, Hiroaki Hosokawa, Tsukasa Fukunaga, Junichi Iwakiri, Goro Terai, Kiyoshi Asai

**Affiliations:** The Department of Computational Biology and Medical Sciences (CBMS), The University of Tokyo, Chiba, 277-8561, Japan; The Department of Computational Biology and Medical Sciences (CBMS), The University of Tokyo, Chiba, 277-8561, Japan; Waseda Institute for Advanced Study, Waseda University, Tokyo, 169-0051, Japan; The Department of Computational Biology and Medical Sciences (CBMS), The University of Tokyo, Chiba, 277-8561, Japan; The Department of Computational Biology and Medical Sciences (CBMS), The University of Tokyo, Chiba, 277-8561, Japan; The Department of Computational Biology and Medical Sciences (CBMS), The University of Tokyo, Chiba, 277-8561, Japan

## Abstract

**Motivation:**

RNA molecules adopt dynamic ensembles of secondary structures, where the local structural context of each nucleotide–such as whether it resides in a stem or a specific type of loop–strongly shapes molecular interactions and regulatory function. Structural-context probabilities therefore provide a more functionally informative view of RNA folding than the minimum free energy structures or base-pairing probabilities. However, existing tools either require $O(N^{3})$ time or employ span-restricted approximations that omit long-range base-pairs, limiting their applicability to large and biologically important RNAs.

**Results:**

We introduce LinearCapR, enabling linear-time, span-unrestricted computation of structural-context marginalized probabilities, using beam-pruned Stochastic Context Free Grammar-based computation. LinearCapR retains global ensemble features lost by span-limited methods and yields superior predictive power on bpRNA-1m(90) dataset, especially for multiloops and exterior regions, as well as long-distance stems. LinearCapR supports analysis of long RNAs, demonstrated on the full genome of SARS-CoV-2. LinearCapR provides the first base-pair-span-unrestricted, linear-time framework for RNA structural-context analysis, retaining key thermodynamic ensemble features essential for functional interpretation. It enables large-scale studies of viral genomes, long non-coding RNAs, and downstream analyses such as RNA-binding protein site prediction.

**Availability and Implementation:**

The source code of LinearCapR is available at https://github.com/hoget157/LinearCapR. The archived software release used in this work is available at Zenodo: https://doi.org/10.5281/zenodo.19450645.

## 1 Introduction

RNA molecules form highly dynamic ensembles of secondary structures rather than a single static conformation. Their conformational states fluctuate according to thermodynamic stability and environmental conditions, making both the determination and prediction of RNA structures a challenging problem. While high-resolution experimental approaches such as NMR spectroscopy, X-ray crystallography, and cryo-electron microscopy can reveal detailed architectures, they are labor- and cost-intensive and do not readily capture alternative conformations at scale.

Computational methods based on nearest-neighbor thermodynamic models provide an efficient alternative. Under the pseudoknot-free assumption, dynamic programming enables the computation of the minimum free energy (MFE) structures (Zuker 1989) or partition functions and base-pairing probabilities (BPPs) (McCaskill 1990) in $O(N^{3})$ time and $O(N^{2})$ space. The ensemble-level quantities offer essential insight into alternative conformations, regulatory switching, and structural accessibility that underlie RNA functions. Within the same ensemble framework, RNA accessibility is often formulated as the probability that a site or segment remains unpaired; it can also be computed globally without a span limit in $O(N^{3})$ time (Bernhart *et al*. 2011).

However, base-pairing information alone is insufficient for many biological questions. The structural context of each nucleotide—such as whether it is located in a stem, hairpin loop, bulge, internal loop, multiloop, or exterior region—strongly influences RNA-binding proteins, catalytic activity of ribozymes, and post-transcriptional regulation. Base-pairing probabilities indicate possible pairing partners for each nucleotide, but they do not directly capture whether that nucleotide is likely to occur in a stem or in a particular loop context, such as a hairpin loop, bulge loop, internal loop, multiloop, or exterior region. Posterior probabilities over these structural contexts therefore provide a richer and more functionally meaningful representation of RNA structural ensemble than BPPs alone. This representation can be more directly relevant to downstream analyses, such as the analysis of RNA-binding protein binding sites, which depend on site accessibility or local structural context rather than on the identity of a specific pairing partner.

CapR introduced a stochastic context free grammar (SCFG)-based framework to compute such structural-context probabilities for each nucleotide (Fukunaga *et al.* 2014), but its original formulation requires $O(N^{3})$ time, limiting its applicability to long RNAs. For practical scalability on long RNAs, CapR and related tools such as RNAplfold and Rfold often impose a base-pair span limit *W*, reducing computational cost to $O(NW^{2})$ (Bernhart *et al.* 2006, Kiryu *et al.* 2008). Yet a span limit necessarily discards long-range base-pairing interactions, which are widespread in natural RNAs—for example, compact viral genomes and end-to-end contacts in mRNAs (Yoffe *et al.* 2011, Lai *et al.* 2018). Thus, efficient and span-unrestricted context profiling has remained elusive.

Beam-search-based linearization provides an effective means of approximating SCFG-based computation in linear time by pruning low-probability states during left-to-right parsing. While related pruning strategies have been explored in other grammar-driven domains (Chiang 2007), the breakthroughs most relevant to RNA biology are LinearFold (Huang *et al.* 2019), LinearPartition (Zhang *et al.* 2020), and LinearTurboFold (Li *et al.* 2021), which established that global ensemble-based RNA structure computation can be approximated with remarkable speed and scalability, even for long viral genomes. Their success provided a new computational paradigm for RNA structure prediction. However, these methods target global folding, partition-function approximation, or homolog-aware structure analysis, rather than direct computation of per-nucleotide structural-context probabilities. Inspired by these developments, we bring beam-pruned computation to a different but complementary objective: computing nucleotide-level structural-context posterior probabilities, which has not previously been addressed by any span-unrestricted or beam-based method.

Here, we introduce LinearCapR, the first linear-time algorithm for computing CapR-style structural-context probabilities without imposing a base-pair span limit. LinearCapR reformulates CapR’s SCFG-based computation within a beam-pruning framework inspired by LinearFold and LinearPartition, enabling efficient inside-outside recursion while preserving long-range base-pairing interactions.

Beam pruning bounds the number of candidate states but does not by itself linearize structural-context computation. LinearCapR therefore introduces constant-time range updates for structural-context profiling, removing residual length dependence and enabling end-to-end linear-time computation.

This advance enables accurate and scalable transcriptome- and genome-wide structural-context analysis, supporting downstream studies that benefit from interpretable, ensemble-aware RNA structure representations.

## 2 Materials and methods

### 2.1 Algorithm

#### 2.1.1 Overview of LinearCapR

LinearCapR approximates CapR’s probabilistic ensemble using a beam-pruned inside-outside dynamic program that scans the sequence from $5^{\prime }$ to $3^{\prime }$. It maintains at most *b* highest-weighted partial structures (“states”) per DP node, pruning low-weight states for reducing search complexity.

CapR linearizes its dynamic programming with time complexity $O(NW^{2})$ by restricting maximum span of base-pairs to a fixed value *W* for an RNA sequence of length *N*. Since LinearCapR imposes no explicit maximum span on base pairs, long-range interactions remain accessible. For a fixed beam width *b* and unpaired-run cap *C*, LinearCapR achieves: **Time:**  $O(Nb^{2}+NbC^{2})$, **Space:**  O(Nb).

#### 2.1.2 Problem formulation

Consider an RNA sequence of length *N* indexed by i=1,…,N. We restrict secondary structures to pseudoknot-free conformations. Each nucleotide *i* belongs to exactly one of the following six structural contexts:

- Stem (S): forms a base pair
- Hairpin loop (H): enclosed by a single base pair
- Bulge (B): in a two-base-pair loop with one-sided unpaired run
- Internal loop (I): in a two-base-pair loop with two-sided unpaired run
- Multiloop (M): enclosed by ≥3 closing base pairs
- Exterior loop (E): not enclosed by any base pair

**Figure 1:**
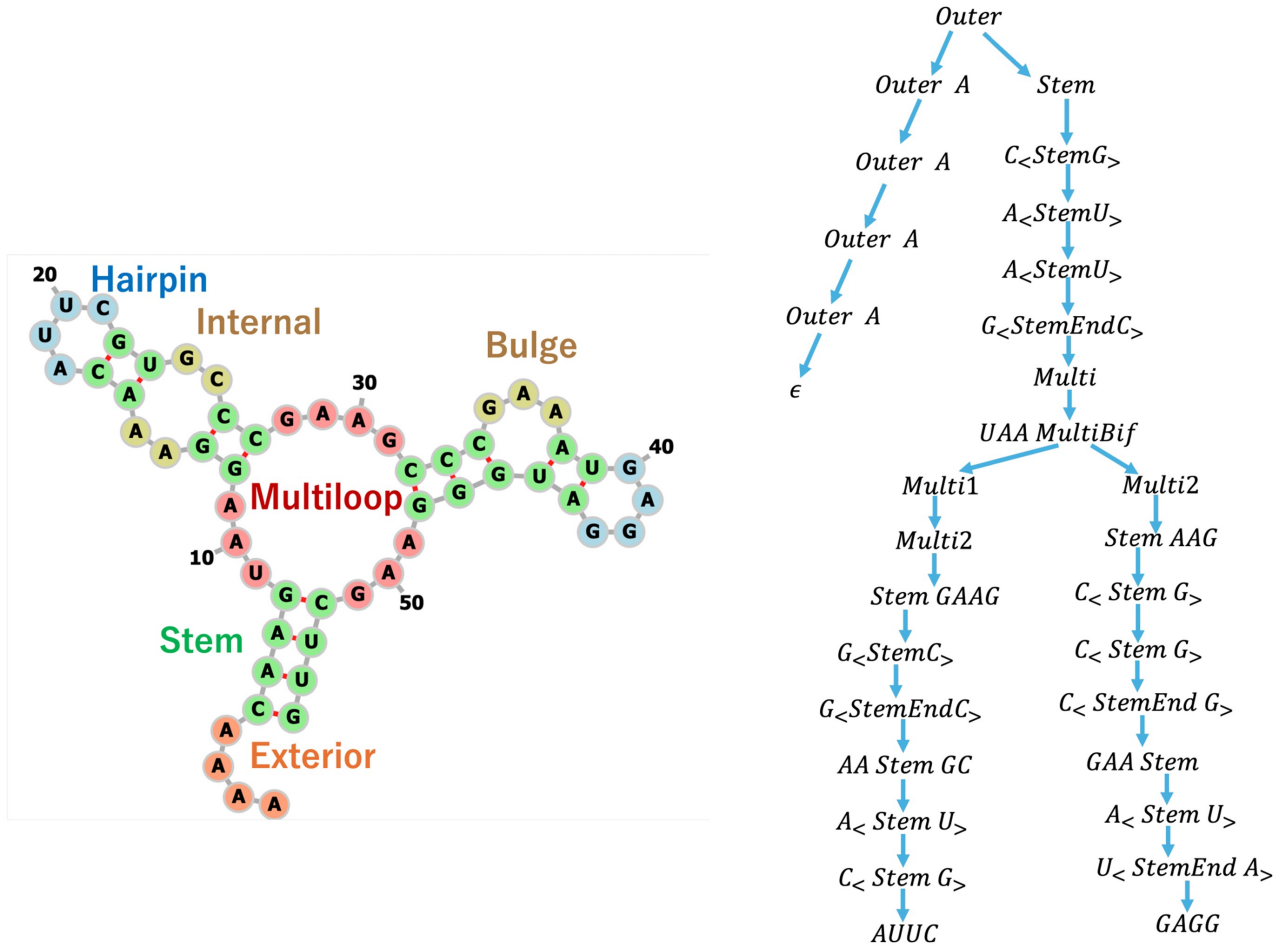
Left: Representative RNA secondary structure annotated with the six structural contexts: stem (S), hairpin (H), bulge (B), internal (I), multiloop (M), and exterior (E). The figure was drawn by Forna (Kerpedjiev *et al.* 2015). Right: Example derivation under the stochastic context-free grammar introduced in the Materials and Methods 2.1.3 (The Grammar and the Algorithm), illustrating how the start symbol Outer is expanded via the Stem, StemEnd, and multiloop production rules to generate the pseudoknot-free structure shown on the left.

Figure 1 illustrates how each nucleotide in the example secondary structure falls into one of the six context categories. The **structural-context profile** is the set of posterior probabilities:

$p(i,\delta )\equiv Pr[x_{i}\;\text{is}\;\text{in}\;\text{context}\;\delta ],\;\text{where}\;\delta \in \{S,H,B,I,M,E\}.$

#### 2.1.3 The grammar and the algorithm

We adopt the stochastic context-free grammar (SCFG) used in CapR (Fukunaga *et al.* 2014) to generate pseudoknot-free RNA secondary structures. The grammar consists of the following seven non-terminal symbols summarized in Table 1.

**Table 1:** Non-terminal symbols used in the SCFG.

Name	Description
Outer	Exterior unpaired region
Stem	Helix extension
StemEnd	Terminates a helix into a hairpin, bulge/internal loop, or multiloop region
Multi	Completed multiloop
MultiBif	Bifurcation into two or more branches
Multi1	One-or-more list of branches
Multi2	One branch followed by a 3′-side run of unpaired bases

The production rules are:


$$\begin{array}{c} \text{Outer}\to \epsilon |\text{Outer}\cdot a|\text{Outer}\cdot \text{Stem}\\ \text{Stem}\to b_{<}\cdot \text{Stem}\cdot b_{>}|b_{<}\cdot \text{StemEnd}\cdot b_{>}\\ \text{StemEnd}\to s_{n}|s_{m}\cdot \text{Stem}\cdot s_{n}(m+n>0)|\text{Multi}\\ \text{Multi}\to s_{n}\cdot \text{MultiBif}\\ \text{MultiBif}\to \text{Multi}1\cdot \text{Multi}2\\ \text{Multi}1\to \text{MultiBif}|\text{Multi}2\\ \text{Multi}2\to \text{Stem}\cdot s_{n} \end{array}$$

where ϵ is the empty string, *a* is a single unpaired nucleotide, $b_{<},b_{>}$ denote the left/right sides of a base pair, and $s_{n}$ represents a run of *n* consecutive unpaired nucleotides. Here, the dot “·” denotes concatenation of symbols. This decomposition mirrors the factorization required by the Inside recursion. The pseudo-code of the algorithm is shown in Algorithm 1, where t(A→B) denotes the Boltzmann weight of production A→B,$\alpha_{s}(i,j)$ ($\beta_{s}(i,j)$) stores the Inside (Outside) score of state *s* spanning indices *i* through *j*. The corresponding Outside pass is provided in Supplementary Algorithm S1.

To enable linear-time pruning we have removed the recursive productions on Multi and Multi2 from the SCFG of Rfold, and bound the number of consecutive unpaired bases by a constant *C*. We impose n≤C and m+n≤C to limit bulge/internal loop sizes and the number of single-strand residues between multibranch helices. Following CapR we set C=30 in all the experiments. Relative to the Rfold grammar, removing the recursion from Multi and Multi2 forces multiloop unpaired segments to be generated directly and shrinks the multiloop recurrences from O(N) candidate transitions to O(C).

Any pseudoknot-free structure whose unpaired runs do not exceed *C* can be generated by this grammar through repeated expansion starting from Outer. Therefore, the grammar provides a complete search space for commonly observed conformations while bounding the fan-out that the beam search needs to explore. The *C* truncation makes the grammar an approximation for the rare loops that exceed 30 contiguous unpaired bases (e.g. when a hairpin, internal, bulge or multiloop consecutive unpaired segment spans tens of nucleotides on one side), but in the bpRNA multiloop regions 99.57% of unpaired runs are 30 nt or shorter (Supplementary Fig. S1), so the approximation leaves only 0.43% of multiloop unpaired stretches outside the search space.

#### 2.1.4 Energy models

We assume that the probability of an RNA adopting a secondary structure σ follows a Boltzmann distribution proportional to exp(−G(σ)/(RT)), where G(σ) is the free-energy of σ, *R* is the gas constant (1.98717 cal/K), and *T* is set to 310.15 K (37°C). Loop energies are evaluated with the Turner2004 or Turner1999 nearest-neighbour parameters (Mathews *et al.* 2004) in this paper. Non-canonical base pairs present in the datasets are treated as unpaired. LinearCapR supports both Turner2004 (default) and Turner1999 parameters via a command-line option. Unless otherwise noted, we report results with Turner2004.

#### 2.1.5 Calculation of structure profile

During the Inside pass, we process sequence positions from 1 to *N* and retain only the top-*b* states for each non-terminal at the current frontier before distributing their scores to successor states.

The pruning threshold is computed by a linear-time selection routine, similar to QuickSelect (Randell and Russell 1963), so that the expected cost of pruning is linear in the beam size. As a result, the overall inside computation runs in $O(Nb^{2}+NbC^{2})$ time and O(Nb) space when the *b* and the *C* are treated as constants.

To avoid numerical underflow, the implementation accumulates values in log-space and applies a polynomial approximation of log_sum_exp following prior work in CONTRAfold (Do *et al.* 2006) and LinearPartition (Zhang *et al.* 2020).

The Outside recursion is performed in reverse order over the surviving Inside states: for each state *s*, we compute $\beta_{s}(i,j)$ only for pairs (i,j) that survived the Inside recursion, which keeps the Outside complexity asymptotically the same as the Inside pass. Algorithm 1 shows the Inside pass, while the complete Outside recursion and full probability aggregation (hairpin, bulge/internal, stem, exterior) are provided in Supplementary Algorithm S1.

Beam pruning alone is not sufficient to fully linearize CapR-style structural-context computation, because structural-context probabilities must be accumulated over contiguous nucleotide ranges for each surviving state. To remove this remaining source of length-dependent cost, LinearCapR introduces constant-time range updates for profile aggregation. Using difference-array buffers, range contributions are recorded without updating every nucleotide position, and the final profiles are recovered by a single prefix-sum pass per context. This avoids explicit per-position updates within each range and keeps the cost of profile aggregation independent of range length, allowing the overall computation to scale effectively linearly.

Algorithm 2 illustrates this strategy for the multiloop case. We use $B_{\text{MultiUnpaired}}$ to denote the Boltzmann-weighted contributions of multiloop unpaired segments. The Multi non-terminal tracks the unpaired segments between the loop’s branches, so p([i,p−1],M) is added only for the unpaired nucleotides, even though the loop is closed by multiple stems. The resulting structure profile satisfies $\sum_{\delta }p(i,\delta )=1$ for every position *i* and provides the desired marginal probabilities for each nucleotide and structural context.

Algorithm 1 Algorithm for the inside algorithm 1: **procedure** Inside algorithm in LinearCapR

2:  **for**  j=1…N  **do** 

3:   $\mathrm{prune}(\alpha_{\text{Stem}},j)$

4:   **for all**  $i\;s.t.\;[i,j]\in \alpha_{\text{Stem}}$  **do** 

5:    $\alpha_{\text{Stem}}(i-1,j+1)+=\alpha_{\text{Stem}}(i,j)\cdot t(\text{Stem}\to$

    $b_{<}\cdot \text{Stem}\cdot b_{>})$ # Stem

6:    $\alpha_{\text{Outer}}(j)+=\alpha_{\text{Outer}}(i-1)\cdot \alpha_{\text{Stem}}(i,j)\cdot t(\text{Outer}\to$

    Outer·Stem)

7:    **for**  n=0…C  **do** 

8:     $\alpha_{\text{Multi}2}(i,j+n)+=\alpha_{\text{Stem}}(i,j)\cdot t(\text{Multi}2\to$

    $\text{Stem}\cdot s_{n})$ # Multiloop

9:    **for all**  p,q s.t. p≤i<j≤q, 0<(i−p)

    +(q−j)≤C  **do** 

10:    $\alpha_{\text{StemEnd}}(p,q)+=\alpha_{\text{Stem}}(i,j)\cdot t(\text{StemEnd}\to$

      $s_{i-p}\cdot \text{Stem}\cdot s_{q-j})$ # Internal/Bulge

11:   $\mathrm{prune}(\alpha_{\text{Multi}2},j)$

12:   **for all**  $i\;s.t.\;[i,j]\in \alpha_{\text{Multi}2}$  **do** 

13:    $\alpha_{\text{Multi}1}(i,j)+=\alpha_{\text{Multi}2}(i,j)\cdot t(\text{Multi}1\to \text{Multi}2)$

14:    **for all**  $k\;s.t.\;[k,i-1]\in \alpha_{\text{Multi}1}$  **do** 

15:     $\alpha_{\text{MultiBif}}(k,j)+=\alpha_{\text{Multi}1}(k,i-1)\cdot \alpha_{\text{Multi}2}(i,j)\cdot$

      t(MultiBif→Multi1·Multi2)

16:   $\mathrm{prune}(\alpha_{\text{MultiBif}},j)$

17:   **for all**  $i\;s.t.\;[i,j]\in \alpha_{\text{MultiBif}}$  **do** 

18:    $\alpha_{\text{Multi}1}(i,j)+=\alpha_{\text{MultiBif}}(i,j)\cdot t(\text{Multi}1\to \text{MultiBif})$

19:    **for**  n=0…C  **do** 

20:     $\alpha_{\text{Multi}}(i-n,j)+=\alpha_{\text{MultiBif}}(i,j)\cdot t(\text{Multi}\to$

      $s_{n}\cdot \text{MultiBif})$ # Multiloop

21:   $\mathrm{prune}(\alpha_{\text{Multi}1},j)$

22:   $\mathrm{prune}(\alpha_{\text{Multi}},j)$

23:   **for all**  $i\;s.t.\;[i,j]\in \alpha_{\text{Multi}}$  **do** 

24:    $\alpha_{\text{StemEnd}}(i,j)+=\alpha_{\text{Multi}}(i,j)\cdot t(\text{StemEnd}\to \text{Multi})$

25:   **for all**  i s.t. j−i−1≤C  **do** 

26:    $\alpha_{\text{StemEnd}}(i,j)+=t(\text{StemEnd}\to s_{j-i-1})\#\text{Hairpin}$

27:   $\mathrm{prune}(\alpha_{\text{StemEnd}},j)$

28:   **for all**  $i\;s.t.\;[i,j]\in \alpha_{\text{StemEnd}}$  **do** 

29:    $\alpha_{\text{Stem}}(i-1,j+1)+=\alpha_{\text{StemEnd}}(i,j)\cdot t(\text{Stem}\to$

    $b_{<}\cdot \text{StemEnd}\cdot b_{>})$ # Stem

30:   $\alpha_{\text{Outer}}(j+1)+=\alpha_{\text{Outer}}(j)\cdot t(\text{Outer}\to \text{Outer}\cdot a)$

    # Exterior

Algorithm 2 Multiloop contributions to the structure profile

1: **procedure** CALC_PROFILE

2:  $Z\leftarrow \alpha_{\text{Outer}}(N)$ # Partition function/normalizer

3:  **for all**  $[p,j]\in \alpha_{\text{MultiBif}}$  **do** 

4:   **for**  i=max(1,p−C)…p−1  **do** 

5:    $p([i,p-1],M)+=\alpha_{\text{MultiBif}}(p,j)\cdot \beta_{\text{Multi}}(i,j)\cdot$

   $B_{\text{MultiUnpaired}}(i,p-1)/Z$

6:  **for all**  $[i,q]\in \alpha_{\text{Stem}}$  **do** 

7:   **for**  j=q+1…min(N,q+C)  **do** 

8:    $p([q+1,j],M)+=\alpha_{\text{Stem}}(i,q)\cdot \beta_{\text{Multi}2}(i,j)\cdot$

   $B_{\text{MultiUnpaired}}(q+1,j)/Z$

The full Outside recursion and the remaining probability updates (hairpin, bulge/internal, stem, exterior) are provided in Supplementary Text S1.

### 2.2 Dataset

1. **bpRNA-1m(90)**: We extracted pseudoknot-free sequences from the bpRNA-1m(90) database (Danaee *et al.* 2018) by selecting entries that belong to Rfam families (Griffiths-Jones *et al.* 2003) and whose annotated structures have page number ≤1 (Clote *et al.* 2012), a criterion that excludes pseudoknots. The resulting subset contains 24 901 sequences with lengths ranging from 11 to 4065 nt, and each with an RNA sequence and an associated reference secondary structure for accuracy evaluation.
2. **RNAcentral**: To stress-test performance on long RNAs, we selected 20 sequences from RNAcentral (RNAcentral Consortium 2021) covering a broad length range (4000–985 945 nt). We divided this range into 20 logarithmically spaced bins and randomly sampled one sequence from each bin (see Supplementary Text S2 for the sampling procedure).
3. **SARS-CoV-2 genome**: We further analysed the 29 903 nt positive-sense RNA genome of SARS-CoV-2 (accession NC_045512) (Rangan *et al.* 2020) to examine how beam width affects performance on a single, moderately long viral genome.

### 2.3 Computational experiments

We designed four complementary sets of experiments to assess (i) runtime and memory usage using bpRNA-1m(90) and RNAcentral datasets, (ii) the accuracy of the structural-context profiles using bpRNA-1m(90), (iii) qualitative long-RNA structure-profile correspondence on RNAcentral candidates, and (iv) the effect of beam width on the partition-function approximation using SARS-CoV-2 genome.

For the accuracy analysis, we obtained ground-truth labels for the six structural contexts by parsing each bpRNA-1m(90) structure with the same SCFG-inspired decomposition used by CapR (Danaee *et al.* 2018). Because we restrict to pseudoknot-free entries, every nucleotide maps unambiguously to exactly one context class, which provides a well-defined basis for the ROC/AUC evaluations.

Benchmark dataset sizes and length ranges are summarized in Supplementary Table S1.

#### 2.3.1 Execution environment

All experiments (bpRNA, RNAcentral, SARS-CoV-2) were run on the same machine: dual-socket Intel Xeon Platinum 8360Y CPUs (72 hardware threads, 2.40 GHz) with 503 GiB RAM under Linux 4.18. The LinearCapR source code was compiled with a GNU C++17 compiler using -O3 -std=c++17 -Wall. No GPU acceleration or multi-node distribution was used.

## 3 Results

### 3.1 Run time and memory usage

To quantify scalability, we measured wall-clock runtime and peak memory usage as a function of sequence length on the bpRNA-1m(90) and on a panel of long RNAs from RNAcentral. Because the time complexity of CapR and LinearCapR depends quadratically on the span limit *W* or beam width *b* (CapR: $\left(O(NW^{2}\right)$; LinearCapR: $O(Nb^{2}+NbC^{2})$)), we varied these parameters over the same range, b,W∈{50,100,200}, and recorded runtime and peak memory for each sequence.

**Figure 2:**
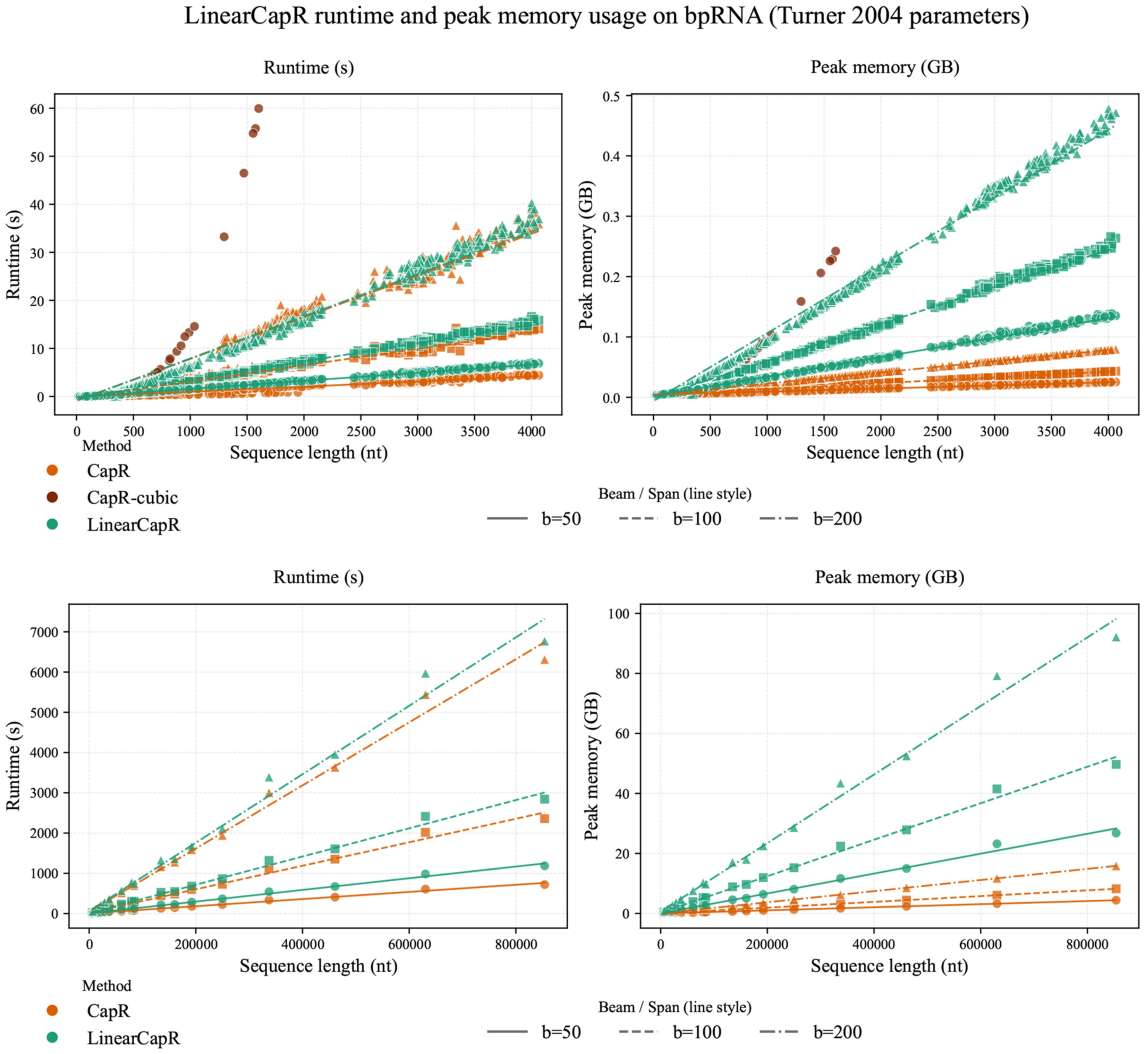
Runtime (top) and peak memory (bottom) versus sequence length for LinearCapR (beam widths b∈{50,100,200}), CapR (span limit W∈{50,100,200}) and CapR-cubic (original cubic-time CapR implementation without span limits). Solid lines denote bpRNA-1m(90) (left panel) and RNAcentral (right panel); each line reflects the per-parameter linear regression fits (one regression per beam width/span limit). CapR-cubic denotes the original $O(N^{3})$ implementation without span constraints, which is impractical for long sequences but is included here as an upper-bound benchmark.

For both methods, runtime and memory increased linearly with length once *W* or *b* was fixed (Fig. 2, left), consistent with the expected O(N) scaling. Runtime differences were modest: CapR exhibits lower runtime (e.g. 7.2 s vs. 12.5 s at 4000 nt when b=W=100) due to its simpler state representation, whereas LinearCapR trades modest additional overhead for span-unrestricted ensemble fidelity. Memory usage showed a clear distinction: LinearCapR retains more active states, resulting in steeper memory growth (3.9GB vs. 1.6GB at b=W=100).

We next evaluated performance on long RNAcentral sequences ranging from 4 kb to 853 kb (Fig. 2, right). Linear scaling was preserved across three order of magnitude in sequence length. For the longest tested sequence (853 910 nt) at b=W=100, CapR required 514 s and 1.65GB, whereas LinearCapR completed 610 s and 10.6GB. Memory demands exceeded 120GB for b≥200, making memory the practical limiting factor for long RNAs.

### 3.2 Accuracy

**Figure 3:**
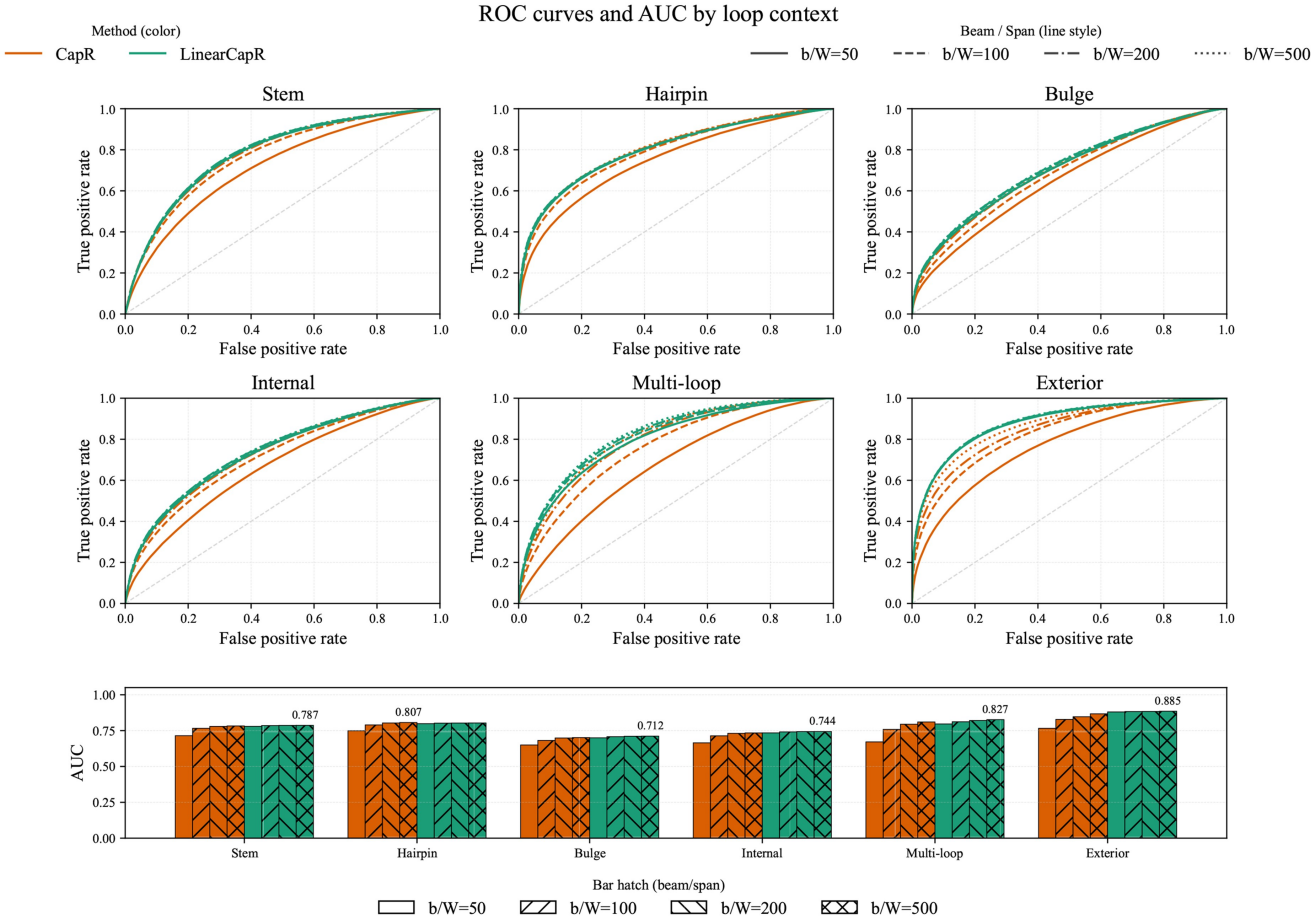
ROC curves for stems (S), hairpins (H), bulges (B), internal loops (I), multiloop (M), and exterior loops (E) comparing LinearCapR and CapR on bpRNA. Bars summarise AUC per context. Larger beam/window sizes improve ROC/AUC for both tools. Exterior loops—strongly influenced by long-range pairs—show the clearest LinearCapR advantage, consistent with its span-unrestricted search.

To assess the discriminative power of the structural-context probabilities, we used the bpRNA-1m(90) with pseudoknot-free reference structures. For each nucleotide i and context δ∈{S,H,B,I,M,E}, the probability p(i,δ) was treated as a one-vs-rest classifier score, and ROC curves and AUC values were computed (Fig. 3).

With b=W=100, LinearCapR improved AUCs across all six contexts, from +0.012 (hairpins) to +0.055 (exterior), where long-range interactions are dominant. Improvements plateaued beyond b≥200. Default parameter comparisons (CapR: Turner1999; LinearCapR: Turner2004) are shown in the main text: Supplementary Fig. S2 provides results with both methods evaluated under Turner1999, where AUCs remain comparable overall, with LinearCapR favoured at small beams and CapR slightly higher at b=W=300.

At b=W=100, ROC curves for LinearCapR dominate those of CapR for every class, yielding AUC gains of 0.019 (stem), 0.012 (hairpin), 0.026 (bulge), 0.027 (internal), 0.053 (multiloop), and 0.055 (exterior). Increasing *b* from 100 to 200 yields <0.003 macro-AUC improvement, indicating that b=200 serves as a practical saturation point for bpRNA-1m(90). Whereas CapR requires spans W>300 to reduce the gap for exterior and multiloop contexts, LinearCapR reaches comparable performance at b=50.

**Figure 4:**
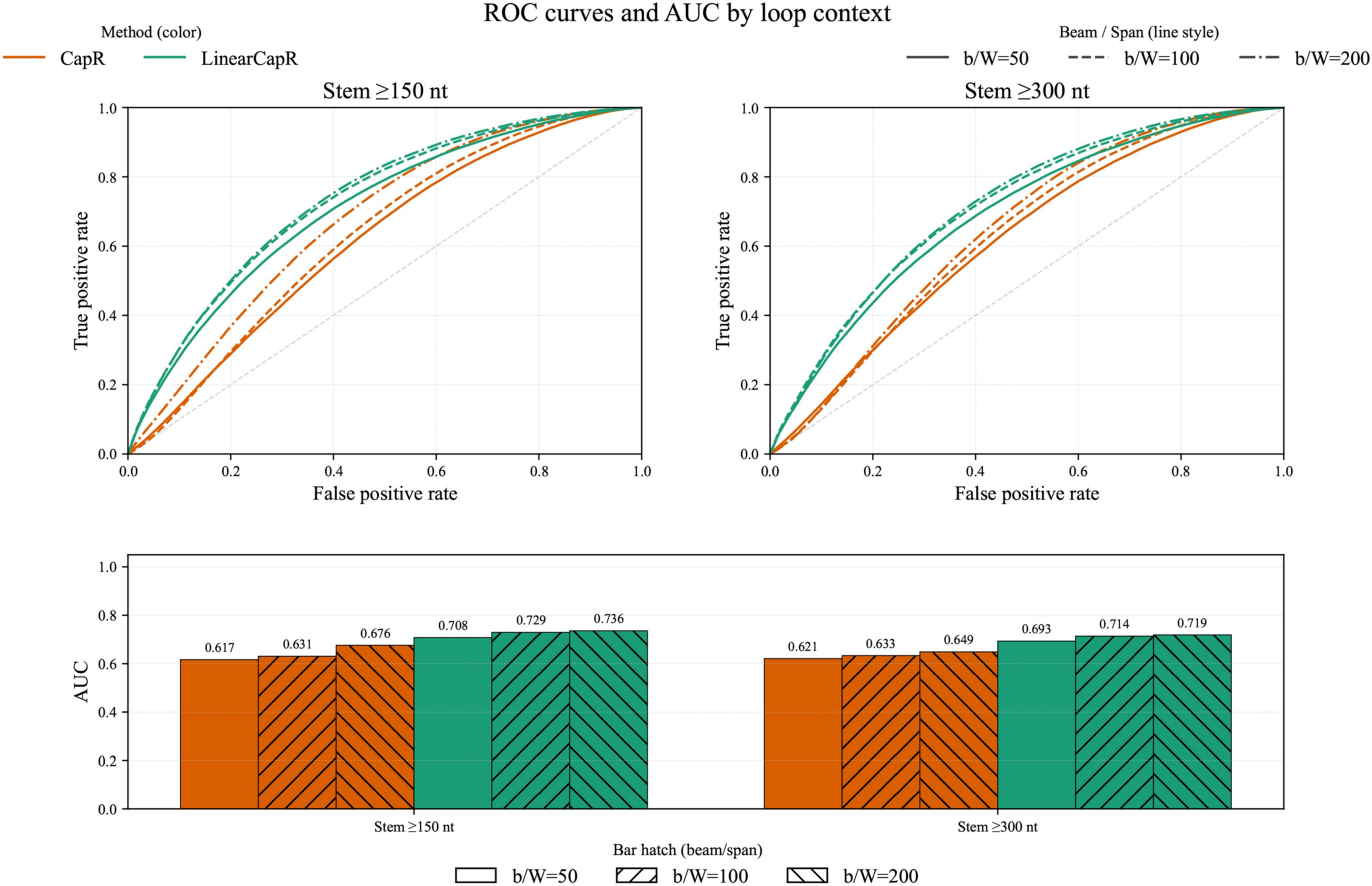
Long-range stem ROC curves and AUC for minimum distances of 150 and 300 nt. Even with a small beam (b=50), LinearCapR surpasses CapR at W=200 for these distant stems.

To directly examine long-range stems, we computed AUC using only stems with pairing distance ≥L∈{150,300} (Fig. 4). LinearCapR showed substantial gains: at b=W=100, AUC increased from 0.617 to 0.716 (L=150) and from 0.633 to 0.714 (L=300). Because CapR cannot represent stems longer than *W*, its performance decreases sharply under these criteria.

This long-range advantage also remained visible when both methods were evaluated under Turner1999 (Supplementary Fig. S3), where LinearCapR generally retained higher AUC for distant stems than CapR across beam/window settings. Together with the corresponding Turner1999 context-level results in Supplementary Fig. S2, this indicates that the main qualitative accuracy trends do not depend strongly on the difference between the Turner2004 and Turner1999 energy parameters.

### 3.3 Qualitative small-RNA structure-profile correspondence

**Figure 5:**
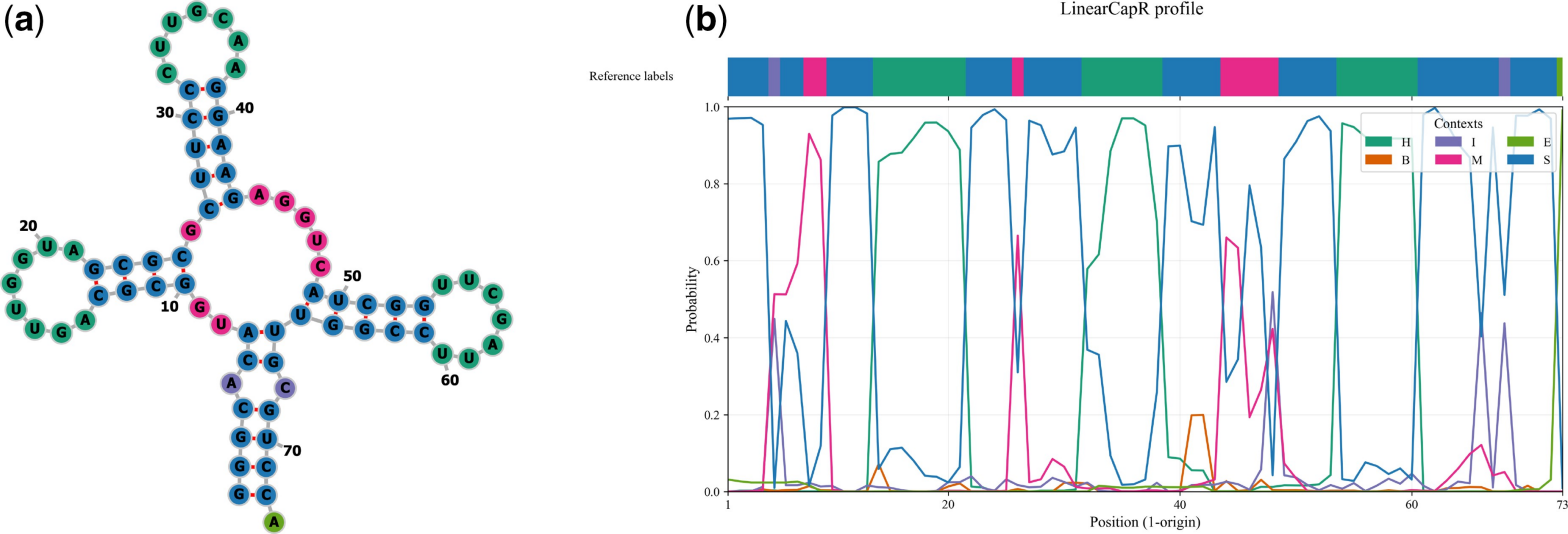
Representative small-RNA example using the yeast alanine tRNA tRNA-Ala-TGC-1–5 from the sacCer3 GtRNAdb/tRNAscan-SE set. (a) Mature reference secondary structure used as a canonical cloverleaf example. (b) LinearCapR profile computed with beam size b=100 for the same sequence, with the reference structure labels shown above the profile. Positions that form stems in the reference structure align with high stem probability, whereas positions in hairpins and other unpaired parts align with higher probabilities for the corresponding non-stem contexts, illustrating qualitative correspondence between the profile and the reference secondary structure.

As a representative small RNA example, we examined a visually interpretable yeast alanine tRNA (tRNA-Ala-TGC-1–5) from the sacCer3 GtRNAdb/tRNAscan-SE set (Chan and Lowe 2009, 2016). We reconstructed the mature reference secondary structure after intron-aware preprocessing and rendered the final diagram with forna (Kerpedjiev *et al.* 2015); the preprocessing details are provided in Supplementary Text S3. This example was selected to provide a visually clear illustration of how the loop profile corresponds to the secondary structure. It was chosen from among candidates with relatively clear agreement, but the same level of agreement is not expected for all RNAs. Figure 5 shows that the LinearCapR profile broadly follows this reference structure: positions that form stems in the reference structure tend to have high stem probability, whereas positions in hairpins and other unpaired parts of the structure tend to have higher probabilities for the corresponding non-stem contexts. In this representative example, the dominant LinearCapR labels, defined here as the highest-probability loop type at each position, agree with the structure-derived labels at 65/73 positions (89.0%), with paired/unpaired agreement at 67/73 positions (91.8%).

### 3.4 Qualitative long-RNA structure-profile correspondence

Since RNAcentral sequences do not provide reference structures, this subsection provides qualitative evidence, while the primary quantitative evaluation is reported in Sect. 3.2.

For long-RNA visualization, we fixed the window length to 120 nt and pre-scanned candidate windows on a single RNAcentral candidate sequence, a maize (*Zea mays*) lncRNA from RNAcentral (URS00023A4DB9; 29 106 nt). We first ran LinearFold on the full-length sequence with beam size b=100, then ranked the resulting 120-nt windows using pre-defined criteria that favored windows having long-range crossing interactions together with multiple context transitions in the profile track. The full window-selection procedure is described in Supplementary Text S4.

**Figure 6:**
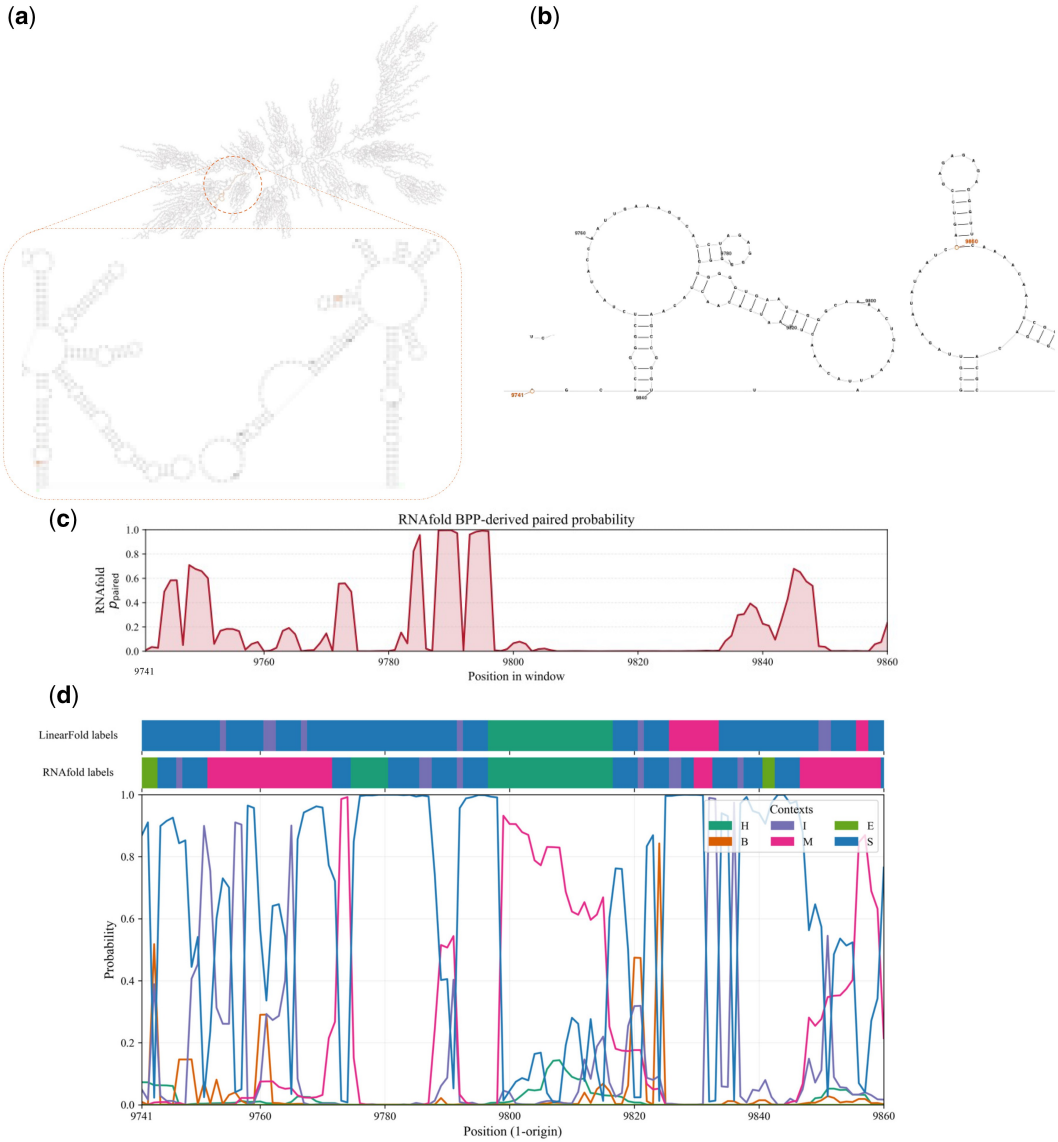
Representative long-RNA window visualization (9741–9860) on URS00023A4DB9 (29 106 nt). Panel (a) consists of two LinearFold-based views: a full-length overview at the top, rendered with Forna (Kerpedjiev *et al.* 2015), and a zoomed local structure for the displayed 120-nt window, rendered with R2DT (McCann *et al.* 2025). The top overview shows the full-length LinearFold prediction with beam size b=100, with the displayed window highlighted. Panel (b) shows an RNAfold structure view for the displayed window, computed with maxBPspan=100, computed on a 320-nt sequence consisting of the displayed window plus 100-nt flanks on both sides. This 320-nt input segment contains all possible pairing partners for the displayed 120-nt region under the span constraint, although it does not reproduce a full-length global fold. Panel (b) was also rendered with R2DT (McCann *et al.* 2025). Panel (c) shows the RNAfold base-pair-probability track for the same 320-nt RNAfold calculation, summarized on the displayed 120-nt window. Panel (d) shows the LinearCapR loop profile on the same 120-nt interval, together with loop-type labelings derived from the two structure views. Together, these panels illustrate that the LinearFold-predicted and RNAfold-predicted structures give different local views of the same region, and that the LinearCapR profile appears qualitatively more similar to the span-unrestricted LinearFold view than to the span-limited RNAfold view.

The selected representative window was 9741–9860. Figure 6 presents four aligned views of this window. Panel (a) shows the span-unrestricted LinearFold prediction with b=100, as a full-length overview together with a zoomed view of the displayed window. Panel (b) shows an RNAfold structure view for the displayed window, computed on the window together with 100-nt flanks on both sides (maxBPspan=100). Panel (c) shows an RNAfold base-pair-probability track summarized on the same window. Panel (d) shows the LinearCapR loop profile for the same 120-nt interval. For the RNAfold comparison, we used a 320-nt sequence consisting of the displayed 120-nt window plus 100-nt flanks on both sides. Under the maxBPspan=100 constraint, this 320-nt sequence contains all possible pairing partners for the central 120-nt region, although it does not reproduce a full-length global fold.

This comparison serves two purposes. First, it examines how the span-unrestricted LinearFold structure and the span-limited RNAfold structure differ for the same region. Second, it shows that the LinearCapR profile more closely matches the span-unrestricted LinearFold-predicted structure, which preserves long-range pairing.

The RNAfold base-pair-probability track provides a pairing-based one-dimensional summary of the same span-limited RNAfold calculation; it is qualitatively related to, but not identical to, the stem-dominant component of the LinearCapR profile. Loop-type labelings derived from the two displayed structures are also shown together with the LinearCapR profile in panel (d). Table 2 summarizes the agreement between these highest-probability LinearCapR labels and the structure-derived labels from the two displayed structure views. In this representative window, the dominant LinearCapR labels appear qualitatively more consistent with the span-unrestricted LinearFold view than with the span-limited RNAfold view, which is reasonable because both LinearCapR and LinearFold avoid an explicit base-pair span limit and can therefore retain the influence of long-range pairing on the local structure. Together, these views suggest that span-unrestricted context profiles retain the influence of long-range pairing that is difficult to preserve under strict local span constraints.

**Table 2:** Agreement between LinearCapR dominant labels and structure-derived labels on the displayed 9741–9860 120-nt window.

Method	Loop type match	Paired/unpaired match
LinearFold beam100	69/120 (57.5%)	89/120 (74.2%)
RNAfold span100	39/120 (32.5%)	64/120 (53.3%)

### 3.5 Beam-width dependence on ensemble quantities

**Figure 7:**
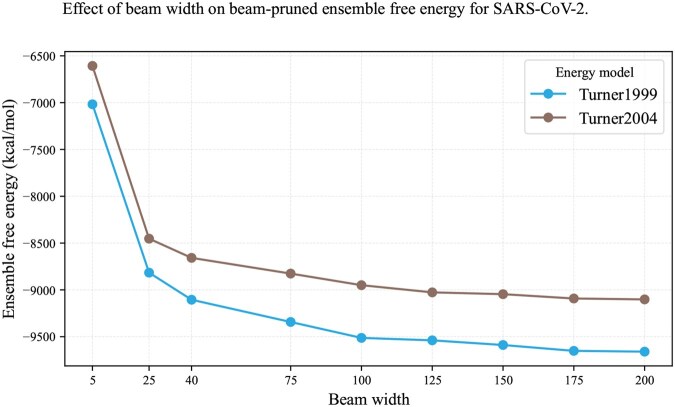
Effect of beam width on ensemble free energy $G_{\text{ensemble}}$ for the SARS-CoV-2 genome. Turner2004 (brown) and Turner1999 (sky blue) curves are overlaid; the latter yield different absolute energies because of the parameters, but both models flatten beyond b≈100. Runtime and memory trajectories are shown in Supplementary Fig. S4.

Finally, we examined how beam width affects both the quality of ensemble estimates and computational cost. We performed beam-width sweeps on the 29 903 nt SARS-CoV-2 genome for *b* ranging from 5 to 200, under both Turner2004 and Turner1999 parameters (Fig. 7). The ensemble free energy $G_{\text{ensemble}}$ in Turner2004 declines noticeably when b<50 but changes little once b≥100; Turner1999 shows the same flattening despite its different absolute values. Runtime and memory scaling for this sweep are provided in Supplementary Fig. S4, and dataset statistics in Supplementary Table S1.

These results indicate that relatively moderate beam width b≥100 already stabilizes ensemble estimates for genome-scale RNAs.

## 4 Discussion

LinearCapR is the first method to compute CapR-style RNA structural-context posterior probabilities in linear time while retaining long-range base-pair interactions. Eliminating the base-pair span limit substantially improves the characterization of exterior and multiloop contexts and enhances detection of long-range stems, which are prevalent in viral RNAs and long noncoding RNAs. Table 3 summarizes key implementation differences between CapR and LinearCapR, including search limits, the time and space complexity, and supported energy parameters.

**Table 3:** Key implementation differences to guide parameter choice.

Aspect	CapR	LinearCapR
Search limit	Span limit*W*	Beam width *b* (no span cap)
Complexity	$O(NW^{2})$	$O(Nb^{2}+NbC^{2})$
Energies	Turner1999	Turner2004/Turner1999

The primary cost of beam pruning is increased memory usage. Sections 3.1–3.3 collectively suggest that beam widths in the range of 100–200 offer a practical trade-off among accuracy, runtime, and memory for transcriptome- and genome-scale RNAs. On the SARS-CoV-2 genome, ensemble thermodynamic estimates stabilize once b≥100, and RNAcentral experiments demonstrate that LinearCapR avoids the bias toward local structures that arises when long-range stems are forcibly truncated.

Future directions include compressed state encodings, GPU parallelization, and adaptive beam control to reduce peak memory demands. Integration with downstream analyses such as RBP-binding prediction or RNA-structure-aware functional annotations represents another promising path.

Overall, LinearCapR enables scalable and biologically meaningful structural profiling of long RNAs with ensemble fidelity. CapR-style context profiles have been shown to improve models of RNA-binding protein specificity from CLIP-seq data. Because LinearCapR scales these profiles to transcriptome- and viral genome-length RNAs without span constraints, it enables systematic re-analysis of existing CLIP datasets and joint modelling of sequence and context preferences. We leave such RBP-focused applications to future work.

*Limitations*: LinearCapR inherits several modelling assumptions from prior SCFG-based RNA folding methods. First, we restrict our search space to pseudoknot-free secondary structures, and non-canonical base pairs are treated as unpaired residues. Second, the grammar bounds contiguous unpaired runs to C=30 nucleotides; this covers 99.57% of multiloop segments in bpRNA (Supplementary Fig. S1) but may underrepresent exceptionally long loops. Third, the beam-pruned inside-outside algorithm yields an approximation of the true Boltzmann ensemble. Our experiments suggest that moderate beam widths (b≈ 100–200) are sufficient for stable ensemble estimates.

## Supplementary Material

btag295_Supplementary_Data

## Data Availability

The source code of LinearCapR is available at https://github.com/hoget157/LinearCapR. The archived software release used in this work is available at Zenodo: https://doi.org/10.5281/zenodo.19450645. The scripts and code used to reproduce the computational experiments are available at https://github.com/TakumiOtagaki/LinearCapR_ComputationalExperiments.

## Supplementary material

Supplementary material is available at *Bioinformatics* online. Supplementary data include all the graphs and tables, and are available at github: https://github.com/TakumiOtagaki/LinearCapR_ComputationalExperiments. The dataset summary is also available, so every figure in the main text can be regenerated from these artifacts hosted in the public repository.
