# Supplementary material for: LinearCapR: linear-time computation of per-nucleotide structural-context probabilities of RNA without base-pair span limits
Source: Bioinformatics. 2026 Jun 15;42(6):btag295. doi: 10.1093/bioinformatics/btag295 (PMC13282080; doi:10.1093/bioinformatics/btag295)
Supplement: btag295_Supplementary_Data [file btag295_supplementary_data.pdf]

Algorithm S1: Detailed algorithms

Here Z denotes the partition function estimated by the Inside pass. The auxiliary terms B_{Hairpin}, B_{Loop}, and $B_{\text{MultiUnpaired}}$ accumulate the Boltzmann-weighted contributions from hairpins, bulge/internal loops, and multibranch-loop unpaired segments, respectively; prefix-sum buffers are used in the multibranch case to aggregate contiguous updates efficiently. The repeated range additions to the structural profile (e.g., $p([i,j],H)\ \mathrel{+}= \ldots$) are implemented via 1D difference-array buffers that record the contributions in $O(1)$ time per range update at the range boundaries and materialize the prefix sum only once per context. This keeps the profile aggregation linear-time despite the nested loops over p, q, and running unpaired segments. This algorithm shows the full Outside recursion that complements Algorithm 1 in the main text.

Algorithm S1.1 Outside algorithm in LinearCapR

procedure CALC_OUTSIDE
  for $j = N \ldots 1$ **do**
    ▷ Outer
    $\beta_{\text{Outer}}(j) \mathrel{+}= \beta_{\text{Outer}}(j+1) \cdot t(\text{Outer} \rightarrow \text{Outer})$
    ▷ Stem
    for all $[i,j] \in \alpha_{\text{Stem}}$ **do**
      $\beta_{\text{Outer}}(i) \mathrel{+}= \beta_{\text{Outer}}(j+1) \cdot \alpha_{\text{Stem}}(i,j) \cdot t(\text{Outer} \rightarrow \text{Outer} \cdot \text{Stem})$
    ▷ StemEnd
    for all $[i,j] \in \alpha_{\text{StemEnd}}$ **do**
      $\beta_{\text{StemEnd}}(i,j) \mathrel{+}= \beta_{\text{Stem}}(i-1,j+1) \cdot t(\text{Stem} \rightarrow \text{StemEnd})$
    ▷ Multi
    for all $[i,j] \in \alpha_{\text{Multi}}$ **do**
      $\beta_{\text{Multi}}(i,j) \mathrel{+}= \beta_{\text{StemEnd}}(i,j) \cdot t(\text{StemEnd} \rightarrow \text{Multi})$
    ▷ MultiBif
    for all $[i,j] \in \alpha_{\text{MultiBif}}$ **do**
      $\beta_{\text{MultiBif}}(i,j) \mathrel{+}= \beta_{\text{Multi1}}(i,j) \cdot t(\text{Multi1} \rightarrow \text{MultiBif})$
      for $n = 0 \ldots C$ **do**
        $\beta_{\text{MultiBif}}(i,j) \mathrel{+}= \beta_{\text{Multi}}(i-n,j) \cdot t(\text{Multi} \rightarrow \text{MultiBif})$
    ▷ Multi2
    for all $[i,j] \in \alpha_{\text{Multi2}}$ **do**
      $\beta_{\text{Multi2}}(i,j) \mathrel{+}= \beta_{\text{Multi1}}(i,j) \cdot t(\text{Multi1} \rightarrow \text{Multi2})$
    for all $[k,i-1] \in \alpha_{\text{Multi1}}$ **do**
      $\beta_{\text{Multi1}}(k,i-1) \mathrel{+}= \alpha_{\text{Multi2}}(i,j) \cdot \beta_{\text{MultiBif}}(k,j) \cdot t(\text{MultiBif} \rightarrow \text{Multi1} \cdot \text{Multi2})$
      $\beta_{\text{Multi2}}(i,j) \mathrel{+}= \alpha_{\text{Multi1}}(k,i-1) \cdot \beta_{\text{MultiBif}}(k,j) \cdot t(\text{MultiBif} \rightarrow \text{Multi1} \cdot \text{Multi2})$
    ▷ Stem
    for all $[i,j] \in \alpha_{\text{Stem}}$ **do**
      $\beta_{\text{Stem}}(i,j) \mathrel{+}= \alpha_{\text{Outer}}(i-1) \cdot \beta_{\text{Outer}}(j+1) \cdot t(\text{Outer} \rightarrow \text{Outer} \cdot \text{Stem})$
      $\beta_{\text{Stem}}(i,j) \mathrel{+}= \beta_{\text{Stem}}(i-1,j+1) \cdot t(\text{Stem} \rightarrow \text{Stem})$
      for $n = 0 \ldots C$ **do**
        $\beta_{\text{Stem}}(i,j) \mathrel{+}= \beta_{\text{Multi2}}(i,j+n) \cdot t(\text{Multi2} \rightarrow \text{Stem})$
      for all $p \le i < j \le q,\ 0 < (i-p)+(q-j) \le C$ **do**
        $\beta_{\text{Stem}}(i,j) \mathrel{+}= \beta_{\text{StemEnd}}(p,q) \cdot t(\text{StemEnd} \rightarrow \text{Stem})$

Algorithm S1.2 Full calculation of structure profile

procedure CALC_PROFILE
  for all $[i,j] \in \alpha_{\text{StemEnd}}$ **do**
    $p([i,j],H) \mathrel{+}= \frac{1}{Z}\,\beta_{\text{StemEnd}}(i,j) \cdot B_{\text{Hairpin}}(i-1,j+1)$
    for $p = i+1 \ldots \min(i+C,\, j-1)$ **do**
      $p([\,i,p-1\,],B) \mathrel{+}= \frac{1}{Z}\,\alpha_{\text{Stem}}(p,j) \cdot \beta_{\text{StemEnd}}(i,j) \cdot B_{\text{Loop}}(i-1,\, j+1,\, p,\, j)$
    for $q = \max(j-C,\, i+1) \ldots j-1$ **do**
      $p([\,q+1,j\,],B) \mathrel{+}= \frac{1}{Z}\,\alpha_{\text{Stem}}(i,q) \cdot \beta_{\text{StemEnd}}(i,j) \cdot B_{\text{Loop}}(i-1,\, j+1,\, i,\, q)$
    for $p = i+1 \ldots \min(i+C,\, j-1)$ **do**
      for $q = \max(p-i+j-C,\, p+1) \ldots j-1$ **do**
        $p([\,i,p-1\,],I) \mathrel{+}= \frac{1}{Z}\,\alpha_{\text{Stem}}(p,q) \cdot \beta_{\text{StemEnd}}(i,j) \cdot B_{\text{Loop}}(i-1,\, j+1,\, p,\, q)$
        $p([\,q+1,j\,],I) \mathrel{+}= \frac{1}{Z}\,\alpha_{\text{Stem}}(p,q) \cdot \beta_{\text{StemEnd}}(i,j) \cdot B_{\text{Loop}}(i-1,\, j+1,\, p,\, q)$
  for all $[p,j] \in \alpha_{\text{MultiBif}}$ **do**
    for $i = \max(1,\, p-C) \ldots p-1$ **do**
      $p([\,i,p-1\,],M) \mathrel{+}= \frac{1}{Z}\,\alpha_{\text{MultiBif}}(p,j) \cdot \beta_{\text{Multi}}(i,j) \cdot B_{\text{MultiUnpaired}}(i,\, p-1)$
  for all $[i,q] \in \alpha_{\text{Stem}}$ **do**
    for $j = q+1 \ldots \min(N,\, q+C)$ **do**
      $p([\,q+1,j\,],M) \mathrel{+}= \frac{1}{Z}\,\alpha_{\text{Stem}}(i,q) \cdot \beta_{\text{Multi2}}(i,j) \cdot B_{\text{MultiUnpaired}}(q+1,\, j)$
  for all $[i,j] \in \alpha_{\text{Stem}}$ **do**
    $p(i,S) \mathrel{+}= \frac{1}{Z}\,\alpha_{\text{Stem}}(i,j) \cdot \beta_{\text{Stem}}(i,j)$
    $p(j,S) \mathrel{+}= \frac{1}{Z}\,\alpha_{\text{Stem}}(i,j) \cdot \beta_{\text{Stem}}(i,j)$
  $p(i,E) = \frac{1}{Z}\,\alpha_{\text{Outer}}(i-1) \cdot \beta_{\text{Outer}}(i+1)$

Fig. S1: Multiloop unpaired run lengths

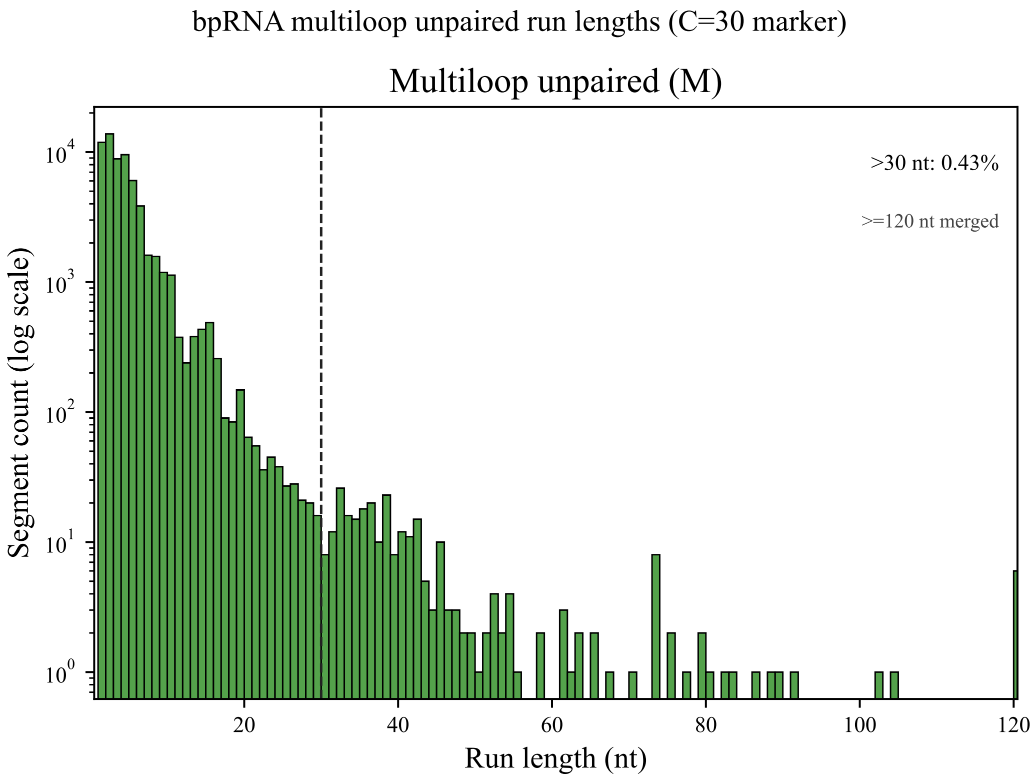

Fig. S1: This figure supports the truncation parameter used in the LinearCapR grammar. It shows the distribution of consecutive unpaired nucleotides within multiloops in bpRNA-1m(90), with a logarithmic y-axis. Only 0.43% of runs exceed 30 nt, indicating that the $C = 30$ cap covers 99.57% of multiloop unpaired segments.

Table S1: Benchmark datasets

Table S1. Overview of the benchmark datasets used in this study. The three sets serve distinct roles: the bpRNA-1m(90) subset is used for accuracy and runtime comparison against reference structures, the RNAcentral set is used for long-RNA scalability assessment, and the SARS-CoV-2 genome is used for beam-width sensitivity analysis.

Dataset	Sequences	Length range	Role in study
bpRNA-1m(90) subset	24,901	11–4,065 nt	Accuracy and runtime comparison
RNAcentral long RNAs	20	4,793–853,910 nt	Scalability assessment
SARS-CoV-2 genome	1	29,903 nt	Beam width sensitivity

Fig. S2: Accuracy with Turner1999 parameters

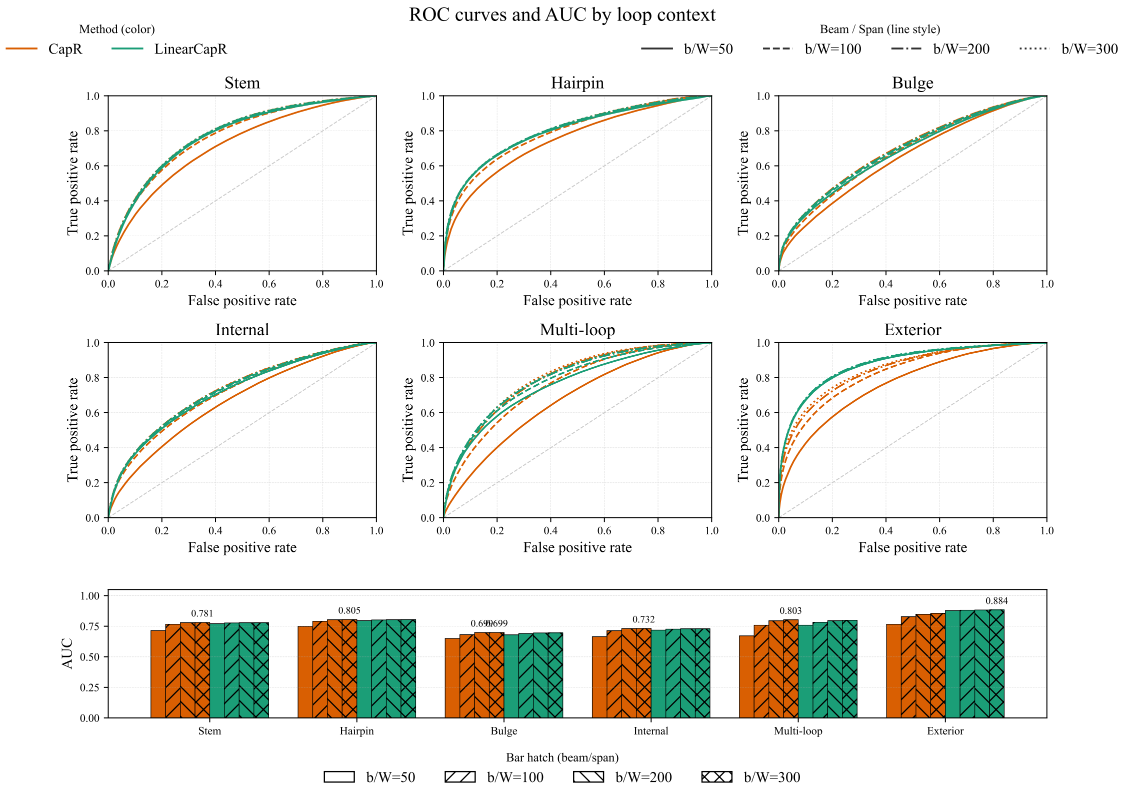

Fig. S2: This figure complements the main-text context-level accuracy comparison (Fig. 3) by showing the same ROC curves and AUC analysis under the Turner1999 setting, with both CapR and LinearCapR evaluated using Turner1999. LinearCapR matches or exceeds CapR for several contexts, especially at smaller beam widths; when the beam/window increases to 300, CapR is slightly higher for many contexts. For exterior loops, LinearCapR yields higher AUC than CapR across all tested beam/window sizes.

Fig. S3: Long-range stems with Turner1999 parameters

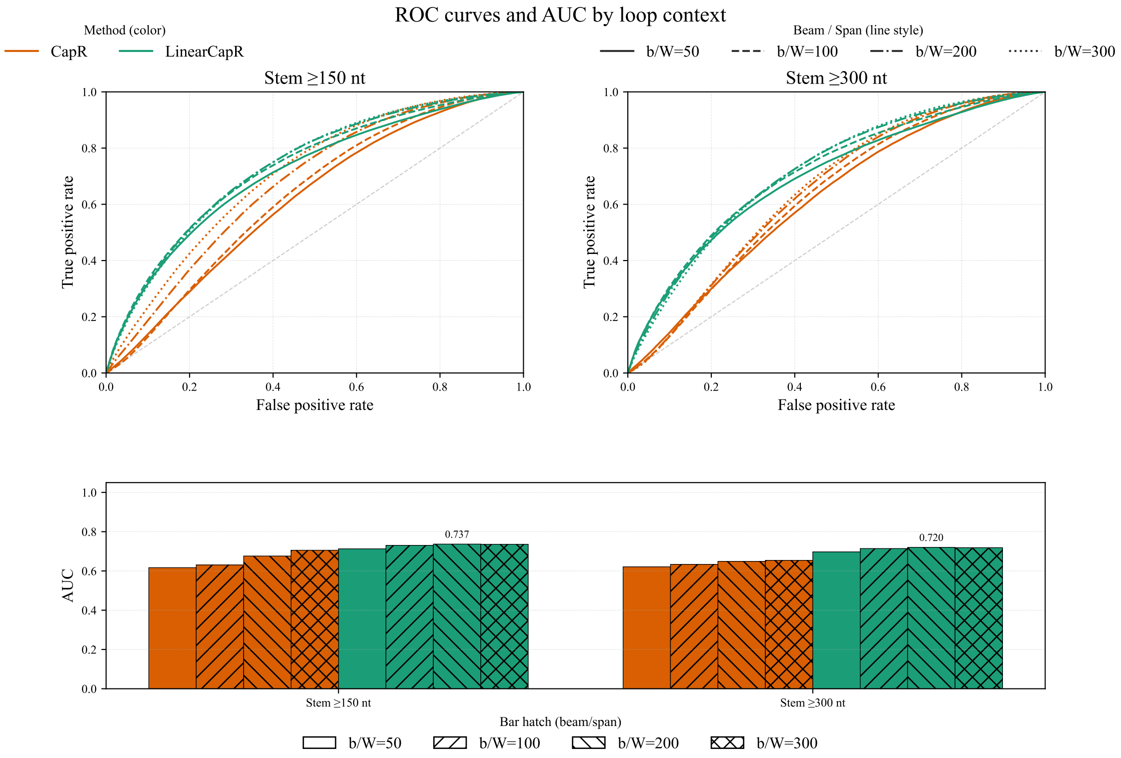

Fig. S3: This figure complements the main-text long-range stem comparison (Fig. 4) by showing the same evaluation under the Turner1999 setting. It shows ROC curves and AUC for long-range stems (minimum distances 150 nt and 300 nt) when both CapR and LinearCapR use Turner1999. LinearCapR generally attains higher AUCs across beam/window settings for these distant stems.

Fig. S4: Beam-sweep runtime and memory

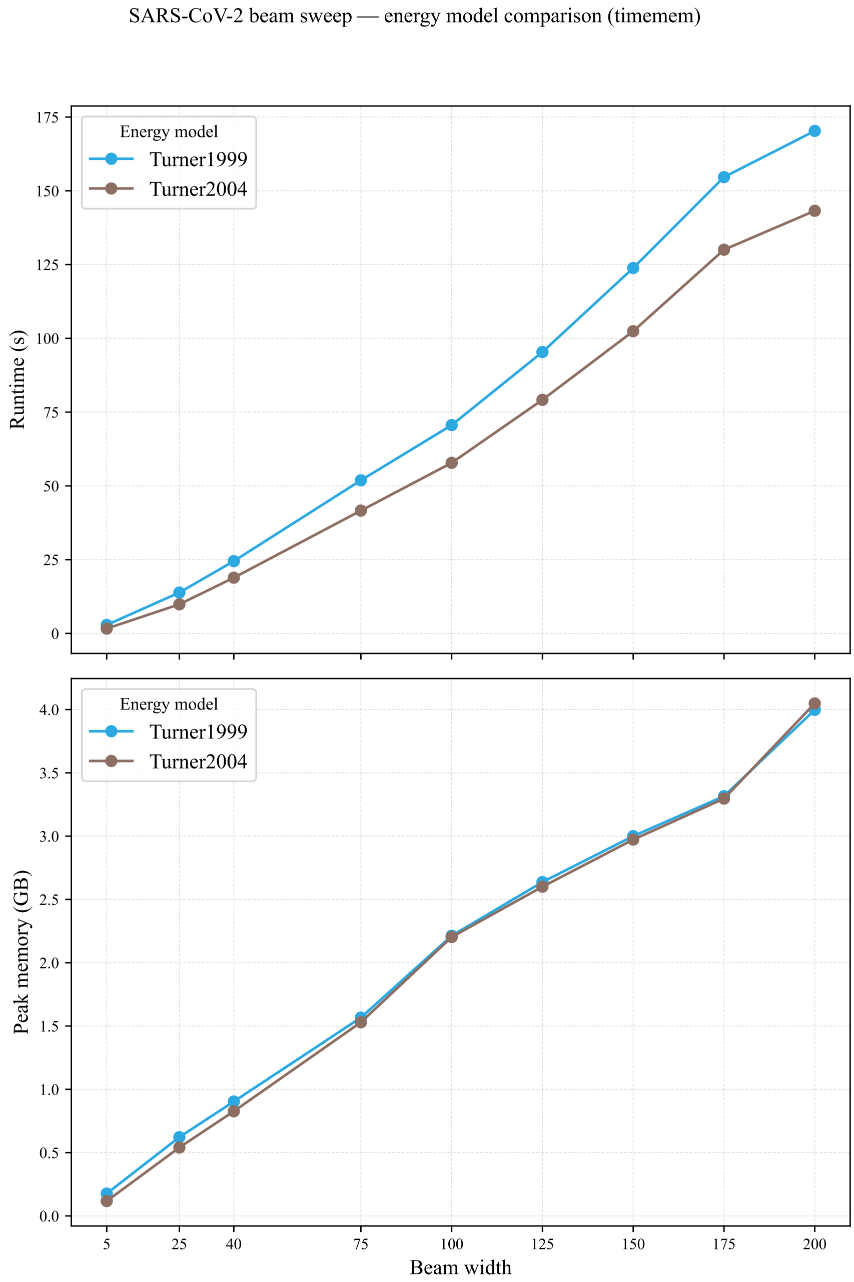

Fig. S4: This figure complements the main-text beam-sweep figure (Fig. 7) by showing runtime and peak memory versus beam width for the SARS-CoV-2 genome under Turner2004 (brown) and Turner1999 (sky blue). Both models scale roughly linearly with b; Turner1999 runs longer but uses similar memory.

Text S2: RNAcentral sampling protocol

This text explains how we assembled the RNAcentral long-RNA set used for the scalability assessment in the main text. The goal was to cover a broad length range without concentrating all examples in one size regime, so we sampled 20 test cases across logarithmically spaced bins of sequence length. The broad range cited in the main text refers to the full RNAcentral source pool considered during this sampling step, whereas Table S1 reports the actual length range of the final 20-sequence sampled set.

Starting from a table of RNAcentral sequence IDs and lengths, we divided the full length range into 20 bins on a logarithmic scale and sampled one sequence from each bin. This produced a set of long RNAs spread across the available size range, rather than concentrating the benchmark in only short, medium, or extremely long sequences. The resulting sample supports the long-RNA scalability analysis in the main text and yields the counts and length ranges reported in Table S1.

A practical implementation of this sampling procedure used a script that reads a tab-separated table with header `name<TAB>length`, constructs 20 log-spaced bins between the minimum and maximum lengths, and samples one entry per bin with a fixed random seed (`seed = 42`, `k = 1`). Bin edges are computed on the log scale and each bin covers $[\,\text{edge}_i, \text{edge}_{i+1} - 1\,]$. The resulting accession-ID list was then used to fetch the sequences, after which summary statistics were compiled for the dataset overview reported in Table S1.

Text S3: Preparation of the tRNA-Ala-TGC-1-5 reference secondary structure

This text documents how the reference secondary structure was prepared for the representative small-RNA example shown in the main text. For that example we used the yeast alanine tRNA `tRNA-Ala-TGC-1-5` from the sacCer3 GtRNAdb/tRNAscan-SE set. The starting data consisted of the mature-sequence FASTA file, the `ss.sort` secondary-structure output, and the tRNAscan summary file reporting intron positions when present.

We converted the tRNAscan secondary-structure notation from `><.` to dot-bracket notation, removed intron segments when needed, and reconstructed the mature reference structure so that its length matched the mature tRNA sequence used as the LinearCapR input. The resulting structure was then mapped to the six structural-context labels used by LinearCapR.

The final two-dimensional rendering of the reference structure was prepared with forna. The corresponding FASTA file, dot-bracket structure, LinearCapR profile, and screening outputs were retained as reproducibility materials for this representative example. These steps explain how the reference structure used in the representative `tRNA-Ala-TGC-1-5` profile-to-structure comparison was obtained.

Text S4: Representative long-RNA window selection procedure

This text explains how we selected the representative long-RNA window used in the main text. The purpose of this procedure was to avoid local-score cherry-picking while still choosing a window in which long-range influence and profile transitions could be interpreted clearly.

For the representative long-RNA visualization in the main text, we used the RNAcentral sequence URS00023A4DB9 (29,106 nt) and fixed the window length to 120 nt. We first ran LinearFold on the full-length sequence with beam size $b = 100$ and then evaluated each 120-nt window using that single span-unrestricted predicted structure.

Let $\mathcal{W}$ denote the set of candidate 120-nt windows. For a window w, let $P_{\text{cross}}(w)$ denote the set of base pairs (i, j) in the full-length LinearFold structure such that exactly one endpoint lies inside w and the other lies outside w. Such pairs cross the window boundary and indicate that the displayed local region may be influenced by long-range pairing outside the shown interval. We summarized this effect by

$$C_{\text{cross}}(w) = \max_{(i,j)\in P_{\text{cross}}(w)} |j - i|,$$

with $C_{\text{cross}}(w) = 0$ when $P_{\text{cross}}(w) = \emptyset$.

To favor windows that were not only long-range-sensitive but also visually interpretable in the profile track, we additionally counted transitions between dominant structural contexts within the same interval. From the dominant context label sequence $\ell_1(w), \ldots, \ell_{120}(w)$, we defined

$$C_{\text{switch}}(w) = \sum_{t=1}^{119} \mathbf{1}[\ell_t(w) \neq \ell_{t+1}(w)].$$

We then used the following two-term score as a pre-defined heuristic to shortlist readable windows, rather than as a theoretically motivated optimality criterion:

$$S(w) = R_{\text{cross}}(w) + R_{\text{switch}}(w),$$

where

$$R_{\text{cross}}(w) = \text{rank}_{\downarrow}(C_{\text{cross}}(w)), \qquad R_{\text{switch}}(w) = \text{rank}_{\downarrow}(C_{\text{switch}}(w)),$$

and $\text{rank}_{\downarrow}$ denotes descending rank over $\mathcal{W}$. In this formulation, C_{cross} was used to retain windows with visible long-range influence, whereas C_{switch} was used only as a readability-oriented heuristic so that the displayed profile was not dominated by a single context over the entire 120-nt interval.

This procedure was intended to avoid local-score cherry-picking while retaining windows that were both sensitive to long-range interactions and readable in the structural-context profile. The final selected window was 9741–9860, which is the representative interval visualized in the main text. A minimal reproducibility bundle was retained for this representative window, including the displayed input files, agreement tables, visualization outputs, and helper scripts.
